# Retraction: Nanocomposite functionalized membranes based on silica nanoparticles cross-linked to electrospun nanofibrous support for arsenic(v) adsorption from contaminated underground water

**DOI:** 10.1039/d5ra90103k

**Published:** 2025-09-12

**Authors:** L. Yohai, H. Giraldo Mejía, R. Procaccini, S. Pellice, K. Laxman Kunjali, J. Dutta, A. Uheida

**Affiliations:** a División Cerámicos, INTEMA, CONICET, UNMdP B7608FDQ Mar del Plata Argentina yohai@fi.mdp.edu.ar; b Functional Materials Group, Department of Applied Physics, KTH Royal Institute of Technology 16440 Kista Stockholm Sweden salam@kth.se

## Abstract

Retraction of ‘Nanocomposite functionalized membranes based on silica nanoparticles cross-linked to electrospun nanofibrous support for arsenic(v) adsorption from contaminated underground water’ by L. Yohai *et al.*, *RSC Adv.*, 2019, **9**, 8280–8289, https://doi.org/10.1039/C8RA09866B.

The Royal Society of Chemistry hereby wholly retracts this *RSC Advances* article due to concerns with the reliability of the data.

The SEM image of mPAN nanofibers in Fig. 2c appears in multiple publications from different research groups in which Abdusalam Uheida is an author.^1–4^

Given the significance of this concern, the Editor has lost confidence that the findings presented in this paper are reliable.

The authors were informed about the retraction of the article. Joydeep Dutta, Karthik Laxman Kunjali, Lucía Yohai, Hugo Giraldo Mejía, Raúl Procaccini and Sergio Pellice have agreed to the retraction. They state that they remain committed to the highest standards of transparency and scientific integrity.

Signed: Joydeep Dutta, Karthik Laxman Kunjali, Lucía Yohai, Hugo Giraldo Mejía, Raúl Procaccini and Sergio Pellice.

Date: 4th September 2025

Retraction endorsed by Laura Fisher, Executive Editor, *RSC Advances*

## References

[cit1] Aziz F., Ouazzani N., Mandi L., Muhammad M., Uheida A. (2016). Sep. Sci. Technol..

[cit2] Yazdi M. G., Ivanic M., Mohamed A., Uheida A. (2018). RSC Adv..

[cit3] Karimiyan H., Uheida A., Hadjmohammadi M., Moein M. M., Abdel-Rehim M. (2019). Talanta.

[cit4] Khalil A., Nasser W. S., Osman T. A., Toprak M. S., Muhammed M., Uheida A. (2019). Environ. Res..

